# Gut microbiota may be involved in Alzheimer’s disease pathology by dysregulating pyrimidine metabolism in APP/PS1 mice

**DOI:** 10.3389/fnagi.2022.967747

**Published:** 2022-08-03

**Authors:** Min Feng, Tianshu Hou, Mingze Zhou, Qiuyu Cen, Ting Yi, Jinfeng Bai, Yun Zeng, Qi Liu, Chengshun Zhang, Yingjun Zhang

**Affiliations:** ^1^School of Rehabilitation Medicine and Healthcare, Hunan University of Medicine, Huaihua, China; ^2^Department of Preventive Traditional Chinese Medicine, Chengdu Integrated TCM, Western Medical Hospital, Chengdu, China; ^3^Health and Rehabilitation School, Chengdu University of Traditional Chinese Medicine, Chengdu, China; ^4^Acupuncture and Tuina School, Shaanxi University of Chinese Medicine, Xianyang, China; ^5^Acupuncture and Tuina School, Chengdu University of Traditional Chinese Medicine, Chengdu, China; ^6^School of Clinical Medicine, Hunan University of Medicine, Huaihua, China

**Keywords:** Alzheimer’s disease, cognitive impairment, gut microbiota, fecal metabolism, 16S rRNA gene sequencing, widely targeted metabolomics, correlation analysis, pyrimidine metabolism

## Abstract

**Introduction:**

Alzheimer’s disease (AD) is the most common form of dementia worldwide. The biological mechanisms underlying the pathogenesis of AD aren’t completely clear. Studies have shown that the gut microbiota could be associated with AD pathogenesis; however, the pathways involved still need to be investigated.

**Aims:**

To explore the possible pathways of the involvement of gut microbiota in AD pathogenesis through metabolites and to identify new AD biomarkers.

**Methods:**

Seven-month-old APP/PS1 mice were used as AD models. The Morris water maze test was used to examine learning and memory ability. 16S rRNA gene sequencing and widely targeted metabolomics were used to identify the gut microbiota composition and fecal metabolic profile, respectively, followed by a combined analysis of microbiomics and metabolomics.

**Results:**

Impaired learning abilities were observed in APP/PS1 mice. Statistically significant changes in the gut microbiota were detected, including a reduction in β-diversity, a higher ratio of *Firmicutes/Bacteroidota*, and multiple differential bacteria. Statistically significant changes in fecal metabolism were also detected, with 40 differential fecal metabolites and perturbations in the pyrimidine metabolism. Approximately 40% of the differential fecal metabolites were markedly associated with the gut microbiota, and the top two bacteria associated with the most differential metabolites were *Bacillus firmus* and *Rikenella*. Deoxycytidine, which causes changes in the pyrimidine metabolic pathway, was significantly correlated with *Clostridium* sp. Culture-27.

**Conclusions:**

Gut microbiota may be involved in the pathological processes associated with cognitive impairment in AD by dysregulating pyrimidine metabolism. *B. firmus*, *Rikenella*, *Clostridium* sp. Culture-27, and deoxyuridine may be important biological markers for AD. Our findings provide new insights into the host-microbe crosstalk in AD pathology and contribute to the discovery of diagnostic markers and therapeutic targets for AD.

## Introduction

Alzheimer’s disease (AD) is the most common neurodegenerative illness in the world, causing cognitive impairment and posing a huge social burden (Elahi and Miller, 2017). There is currently no effective treatment for this condition. Accumulation of β-amyloid plaques and hyperphosphorylation of tau protein in the brain are considered two typical neuropathological hallmarks of AD. However, drugs targeting β-amyloid have failed to show clinical benefits (Vaz and Silvestre, 2020), suggesting that multiple factors are involved in disease onset and progression.

Recent studies have shown that gut bacteria also participate in AD pathogenesis. Researchers have observed gut bacterial alterations in both AD mice and human patients. In APP/PS1 mice, gut bacterial dysbiosis precedes cerebral amyloidosis (Chen et al., 2020). Furthermore, AD-like pathogenesis in APP/PS1 mice can be alleviated by fecal transplantation (Sun et al., 2019), and altering the gut microbiota could provide benefits for patients with AD (Wu L. et al., 2021). These findings imply that gut microbiota alterations may be independent of other known AD biomarkers.

Microorganisms affect the host mainly through their metabolites (Saji et al., 2019). Evidence suggests that small molecule metabolites play an important role in AD development, including microbiome-related metabolites (Ticinesi et al., 2019). Metabolomics provides an excellent tool to investigate the interplay of the host-gut microbiota, and the fecal metabolome is certainly a valuable source for understanding how microbiome-related metabolites are involved in the disease. Widely targeted metabolomics technology offers the advantages of breadth, precision, and high-throughput characteristics, enabling rapid and accurate detection of a wider range of metabolites (Tian et al., 2020), and contributes to the discovery of a new metabolic spectrum. Consequently, it is superior in studying the pathogenesis of multi-etiological diseases, such as AD (González-Domínguez et al., 2018). However, this new technique has not been used to study the host metabolic phenotypic changes induced by gut microbes in AD.

The combined analysis of two core technologies in colon research, microbiomics and metabolomics, can elucidate the co-occurrence of microbes and metabolites and explore the association between microbes and human diseases more comprehensively (Knight et al., 2018). In this study, we first employed 16S rRNA gene sequencing and ultra-high performance liquid chromatography-tandem mass spectrometry (UPLC-MS/MS)-based widely targeted metabolomics to characterize gut bacterial communities and fecal metabolic profiles of APP/PS1 double-transgenic mice. Secondly, we performed a correlation analysis of microbiota and metabolites to explore pathways by which gut microbiota are involved in AD pathogenesis through its metabolites and to probe novel AD markers.

## Materials and methods

### Animals

Eight male APP/PS1 double-transgenic mice aged 7 months were used as animal models of AD (group A). As controls, eight sex-and age-matched wild-type C57BL/6J mice were employed (group C). SPF grade mice were purchased from Zhishan Institute of Health Medicine Co., Ltd. [Beijing, China; license number: SCXK (Su) 2016-0010]. Two mice were housed per cage in standard conditions (21 ± 2°, 50 ± 5% humidity, and 12 h light/dark cycle) with free access to food and water. Animal experimental procedures and designs were approved by the Ethics Committee of Hunan University of Medicine (No. 2019A03001) and were performed in accordance with the National Institutes of Health Guide for the Care and Use of Laboratory Animals.

### Morris water maze test

The Morris water maze (MWM) test was conducted in a pool with an 80 cm diameter (Techman, Chengdu, China) (Wang et al., 2019) to evaluate the spatial learning and memory abilities of the mice. A mix of skimmed milk powder and water was added until it rose to 1 cm above the height of the platform, which had a diameter of 10 cm. The temperature of the mixture was maintained at 20 ± 1°C. The water maze was divided into four equal quadrants, and four starting locations were selected. A blue curtain with four fixed signs of different shapes surrounded it. The test consisted of three procedures: cued learning, a place navigation test, and a spatial probe test. Cued learning consisting of four trials was performed the day before the place navigation test to detect the ability of the mice to learn to swim to a cued target and adapt to the environment. In cued learning, the hidden platform was marked by a “flag” above the water surface (Vorhees and Williams, 2006). The place navigation test was carried out over the next 5 days, with four trials per day. The mice were placed in the water to start from four different locations, and the time taken to locate and climb to the hidden platform within 90 s was recorded as the escape latency. Mice that failed to locate the platform within 90 s were guided to the platform and left to remain there for 10 s with an escape latency recorded as 90 s. 24 h after the last trial of the place navigation test, a spatial probe test was carried out, in which the platform was removed. Mice were placed in the water from the starting location opposite to the original platform quadrant, and they were given 60 s to swim. The number of platform-site crossovers and effective area crossovers, percent time and percent distance in the target quadrant, and latency to first target-site crossover within 60 s were recorded.

### Sample collection

Fresh fecal samples (*n* = 8/group) were collected into individual sterile EP tubes on two consecutive mornings (Wang et al., 2019) after the MWM test and immediately frozen at −80°C until analysis (Giridharan et al., 2022).

### Gut microbiome analysis

#### 16S rRNA gene sequencing

Total genomic DNA (gDNA) was collected from each fecal sample using the CTAB method (Li et al., 2020). Approximate DNA concentration and purity were monitored using 1% agarose gel electrophoresis. DNA was diluted to 1 ng/μL with sterile water depending on the concentration. The V4 regions of the bacterial 16S rRNA gene were PCR-amplified as 515F (5′-GTGCCAGCMGCCGCGGTAA-3′) and 806R (5′-GGACTACHVGGGTWTCTAAT-3′) from gDNA. The same volume of 1× loading buffer (containing SYB green) was mixed with PCR products and detected by electrophoresis on a 2% agarose gel. The Qiagen Gel Extraction Kit (Qiagen, Germany) was used for the purification after the PCR products were mixed in equidensity ratios. A TruSeq^®^ DNA PCR-Free Sample Preparation Kit (Illumina, United States) was used for generating sequencing libraries. A Qubit@ 2.0 Fluorometer (Thermo Scientific) and Agilent Bioanalyzer 2100 system were used to evaluate the quality of the library. Finally, the library was sequenced using an Illumina NovaSeq platform, and paired-end reads of 250 bp were produced.

#### Data analysis

Uparse software (Uparse v7.0.1001) was used for sequencing data analysis. Sequences with ≥97% similarity were assigned to the same operational taxonomic unit (OTUs). For each representative sequence, the Silva database was used based on the Mothur algorithm to annotate taxonomic information. To investigate the phylogenetic relationship of different OTUs and the differences in the dominant bacteria in different groups, multiple sequence alignments were carried out with the MUSCLE software (version 3.8.31). A standard sequence number corresponding to the sample with the least number of sequences was used to normalize the OTUs abundance information. α and β diversity analyses were carried out, based on the normalized data. The Chao1, Shannon, and ACE indices were used to measure α diversity. These indices were computed with QIIME (version 1.7.0) and shown with R software (version 2.15.3). With QIIME (version 1.9.1), β diversity was determined for both weighted and unweighted UniFrac. Principal coordinate analysis (PCoA) was carried out to obtain the principal coordinates and visualize complex multidimensional data. The WGCNA package, stat packages, and ggplot2 package in R software (version 2.15.3) were used for PCoA analysis. Linear discriminant analysis (LDA) effect sizes (LEfSe) were used for supervised comparisons of microbiota between groups. Important taxonomic differences between the two groups were determined by Log LDA score > 4.0 and *P* < 0.05. Function prediction was performed using Tax4Fun (Gresse et al., 2019).

### Fecal metabolomics analysis

#### Sample preparation and extraction

Fecal samples were thawed on ice. One sample of 50 mg (±1 mg) was homogenized with 500 μL ice-cold methanol/water (70%, v/v) with internal standard as a reference for quantification. The sample was vortexed for 3 min, sonicated in an ice-water bath for 10 min, vortexed again for 1 min, and centrifuged at 12,000 rpm for 10 min at 4°C. Then, 250 μL of the supernatant was taken in a tube and centrifuged at 12,000 rpm for another 5 min at 4°C. Subsequently, 150 μL of the supernatant was added to the liner of the corresponding injection vial for further analysis.

#### Ultra-high performance liquid chromatography conditions

The LC-ESI-MS/MS system (UPLC, ExionLC AD; MS, QTRAP^®^ System) was used to analyze the sample extracts. The following were the parameters of the analysis: the UPLC column was a Waters ACQUITY UPLC HSS T3 C18 (1.8 μm, 2.1 mm*100 mm); column temperature was 40°C; flow rate was 0.4 mL/min; injection volume was 2 or 5 μL; solvent system was water (0.1% formic acid) and acetonitrile (0.1% formic acid); gradient program was 95:5 V/V at 0 min, 10:90 V/V at 10.0 min, 10:90 V/V at 11.0 min, 95:5 V/V at 11.1 min, 95:5 V/V at 14.0 min.

#### QTOF-MS/MS

In order to collect MS/MS spectra on an information-dependent basis (IDA), a triple TOF mass spectrometer was employed. In this mode, the acquisition software (TripleTOF 6600, AB SCIEX) performs a continuous evaluation of the MS data obtained from the full-scan survey during the acquisition process. Based on preselected criteria, the program then activates the acquisition of MS/MS spectra. In each cycle, 12 precursor ions with intensities greater than 100 were selected for fragmentation using a collision energy of 30 V. The following were the ESI source conditions: ion source gas 1, 50 psi; ion source gas 2, 50 psi; curtain gas, 25 psi; source temperature, 500°C; and ion spray voltage floating at 5,500 V in positive and −4,500 V in negative mode.

#### ESI-Q TRAP-MS/MS

On a triple quadrupole linear ion trap mass spectrometer (QTRAP), LIT and triple quadrupole (QQQ) scans were obtained. The QTRAP^®^ LC-MS/MS system was equipped with an ESI turbo Ion-Spray interface operating in positive and negative ion mode, and controlled by Analyst software 1.6.3 (Sciex). The following were the ESI source operating parameters: the source temperature was 500°C; the ion spray voltage was 5,500 V (positive) and −4,500 V (negative); the ion source gas I, gas II, and curtain gas were set at 50, 50, and 25 psi, respectively; the collision gas was high. Instrument tuning and mass calibration were carried out using solutions containing 10 and 100 μmol/L of polypropylene glycol, respectively. A specific set of MRM transitions was monitored based on the metabolites eluted during each period.

### Metabolite identification and Kyoto Encyclopedia of Genes and Genomes pathway analysis

Metabolite data were unit variance scaled and then an unsupervised principal component analysis (PCA) was performed using the statistics function prcomp within R (version 3.5.0, base package). Using the R package ComplexHeatmap, a hierarchical cluster analysis (HCA) was carried out, and the results are presented as heatmaps with dendrograms. The color spectrum represented the normalized signal intensities (unit variance scaling) of the metabolites. Variable importance in projection (VIP) values were obtained from the OPLS-DA results and the score plots were created by the R package MetaboAnalystR. Before OPLS-DA, the data were log-transformed (log_2_) and mean-centered. In order to prevent overfitting, a test consisting of 200 permutations was carried out. VIP ≥ 1, fold-change value ≥ 2 or ≤0.5, and *P* < 0.05 were used to identify differential metabolites between the groups. The differential metabolites were annotated using the Kyoto Encyclopedia of Genes and Genomes (KEGG) compound database and mapped to the KEGG pathway database. For a given list of metabolites, *p*-values from hypergeometric tests were used to determine the degree of significant pathway enrichment.

### Statistical analysis

Data analysis was carried out with SPSS 17.0. Data obtained from the place navigation test were analyzed using repeated-measures analysis of variance (ANOVA), the process of the general linear model in SPSS. The independent samples *t*-test or Wilcoxon’s test were used to compare the data between the two groups. Spearman’s correlation test was used to investigate the correlations between gut microbes and metabolites. GraphPad Prism 5 software (GraphPad Software, San Diego, CA, United States) was used to create graphical representations. Data are expressed as mean ± standard error of the mean (SEM), unless stated otherwise. Statistical significance was set at *P* < 0.05.

## Results

### Morris water maze

**FIGURE 1:**
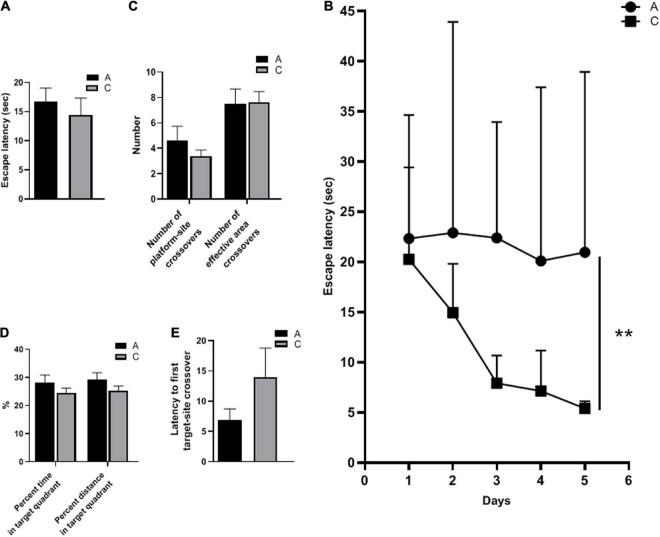
Spatial learning and memory abilities of APP/PS1 and wild-type mice in the MWM test. Escape latency in cued learning **(A)** and place navigation test (mean ± standard deviation) **(B)**, the number of platform-site crossovers and effective area crossovers **(C)**, percent time and percent distance in the target quadrant **(D)**, and latency to first target-site crossover **(E)** in the spatial probe test. Groups: A, APP/PS1 double-transgenic mice; C, wild-type control group of C57BL/6J mice; *n* = 8/group. **P < 0.01.

The spatial learning ability of the mice was evaluated by escape latency. There was no significant difference in escape latency between APP/PS1 and WT mice during cued learning (*P* = 0.543, Figure 1A), indicating that all mice could complete the test with normal motor and visual function. In contrast, in the place navigation test, escape latencies of APP/PS1 mice were significantly longer (*P* = 0.004, Figure 1B). The number of platform-site crossovers and effective area crossovers was used to assess the spatial memory abilities of the mice. These two parameters did not differ significantly between the groups (*P* = 0.324, *P* = 0.933, Figure 1C). Furthermore, no significant differences were observed in other parameters of the spatial probe test, including percent time and distance in the target quadrant (*P* = 0.240, *P* = 0.235, Figure 1D), and latency to first target-site crossover (*P* = 0.203, Figure 1E).

### Gut microbiome

#### Community richness and diversity of the gut microbiota

**FIGURE 2:**
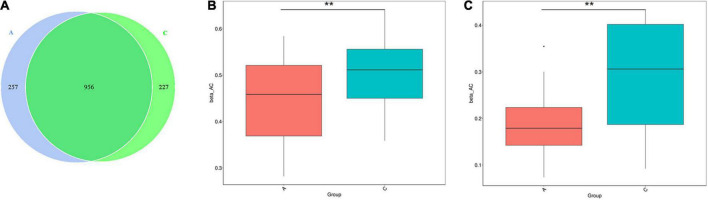
Gut microbiota diversity in APP/PS1 and wild-type mice. The Venn diagram illustrates the overlap of the OTUs identified in gut microbiota between the two groups **(A)**. β-diversity of the gut microbiota between the two groups according to t-test of unweighted **(B)** and weighted **(C)** UniFrac distance. Groups: A, APP/PS1 double-transgenic mice; C, wild-type control group of C57BL/6J mice; *n* = 8/group. **P < 0.01.

16S rRNA gene sequencing performed on the fecal samples was used to assess the gut microbiota of mice. As shown in the Venn diagram, 1,440 OTUs were identified, of which 257 unique OTUs belonged to APP/PS1 mice (Figure 2A). α diversity was applied to analyze the complexity of the gut microbiota diversity in a sample. The Chao1 and ACE indices were selected to identify community richness, and the Shannon index was used to identify community diversity. There were no apparent differences in these indices between the two groups (data not shown). β-diversity was applied to analyze differences in the gut microbiota with respect to species complexity. The results of both unweighted and weighted UniFrac distance analysis showed a significant decrease in β-diversity in APP/PS1 mice (*P* = 0.004, *P* = 0.00, Figures 2B,C).

#### Composition of the gut microbiota

**FIGURE 3:**
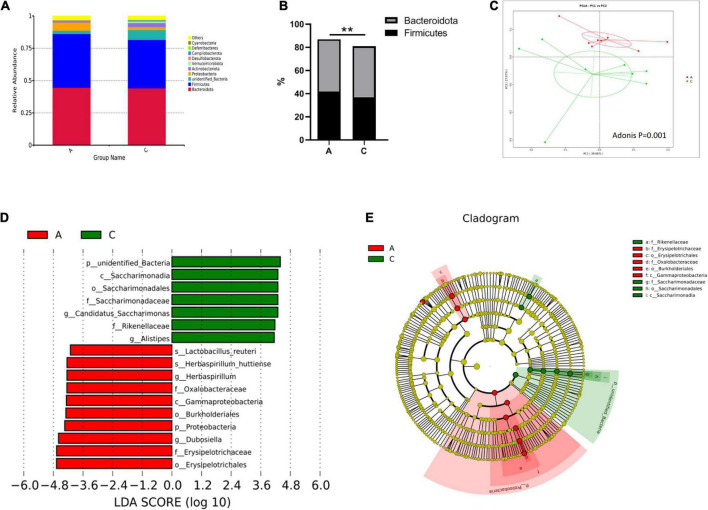
Gut microbiota composition in APP/PS1 and wild-type mice. Relative abundance stacked barplots at the phylum level **(A)** and the *Firmicutes/Bacteroidota* ratios **(B)**. PCoA based on the weighted UniFrac phylogenetic distance is compared between APP/PS1 and wild-type mice (adonis *P* < 0.01) **(C)**. Comparison of the representative taxonomic abundance between the two groups identified by LEfSe **(D)**. Cladogram of the taxa enriched in the gut microbiota **(E)**. The central point represents the root of the tree (bacteria), and the ring represents the taxonomic level (phylum to genus). The diameter of the ring represents the relative abundance of that taxon. Groups: A, APP/PS1 double-transgenic mice; C, wild-type control group of C57BL/6J mice; *n* = 8/group. **P < 0.01.

Overall microbial composition was evaluated at different taxonomic levels. *Firmicutes* and *Bacteroidota* were the predominant phyla in the mouse gut microbiome, with percentages of approximately 40 (Figure 3A). However, in APP/PS1 mice, the *Firmicutes/Bacteroidota* ratio was notably higher compared with WT mice (0.935 vs. 0.850, *P* = 0.000, Figure 3B). As shown in Figure 3C, the two groups were successfully separated in the PCoA score plot, with the PC1 and PC2 principal components explaining 39.08 and 27.07% of the variation, respectively. Furthermore, the permutational MANOVA *p*-value (calculated by the adonis function in the vegan R package) was 0.001.

Metastatic analysis was performed to determine the differences in the microorganisms between the groups. The results showed that 4, 6, 22, 37, 67, and 35 microbes were markedly altered in APP/PS1 mice at the phylum, class, order, family, genus, and species levels, respectively. A log LDA score > 4.0, *P* < 0.05, was used to identify important taxonomic differences between the groups, and a representative cladogram was used to show the variant taxa at different taxonomic levels. Figure 3D shows the top 17 taxonomic differences, with the relative abundance of 7 bacteria significantly lower and 10 bacteria significantly higher in APP/PS1 mice. The cladogram showed the phylogenetic distribution of the gut microbiota associated with the two groups (Figure 3E).

#### Prediction of microbial community function

**FIGURE 4:**
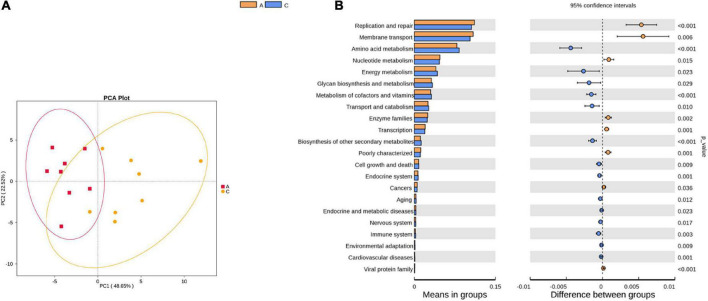
Principal component analysis (PCA) plot of microbial community function prediction at level 2 in APP/PS1 and wild-type mice **(A)** and comparative plots of significantly different pathways **(B)**. Groups: A, APP/PS1 double-transgenic mice; C, wild-type control group of C57BL/6J mice; *n* = 8/group.

Tax4Fun was used to predict the functional genes of the gut microbiota based on the 16S rRNA gene sequencing results. From level 1 to level 3, extensive changes in microbiota function were observed in APP/PS1 mice. For instance, at level 2, the predicted functions of the two groups were partly separated in the PCA plot (Figure 4A), and multiple pathways were significantly different between the two groups (Figure 4B), including amino acid metabolism, nucleotide metabolism, energy metabolism, metabolism of cofactors and vitamins, aging, nervous system, and immune system.

### Fecal metabolome

#### Fecal metabolic profiles

**FIGURE 5:**
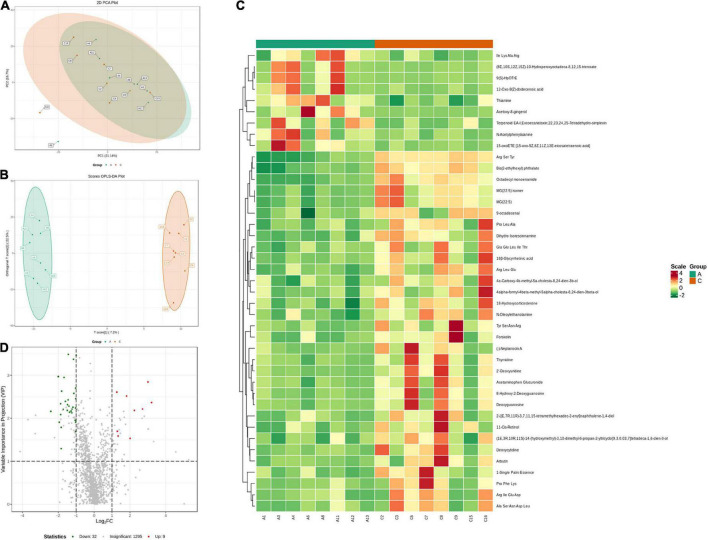
Variation characteristics of fecal metabolites and screening for differential metabolites in APP/PS1 and wild-type mice. PCA plot **(A)** and score plot derived from the OPLS-DA model **(B)**. Heatmap **(C)** and volcano plots **(D)** of differential metabolites between the groups (red, upregulated in APP/PS1 mice; green, downregulated in APP/PS1 mice). Groups: A, APP/PS1 double-transgenic mice; C, wild-type control group of C57BL/6J mice; *n* = 8/group.

Fecal metabolic profiles were acquired using UPLC-MS/MS. As shown in the PCA plot, the fecal metabolites of the two groups were not well-separated, and no visible clustering trend was observed between the groups (Figure 5A). The OPLS-DA model was used to analyze the compounds that led to the differences between the groups. The OPLS-DA scores showed that the two groups were scattered in two different regions, with R2 (Y), R2 (X), and *Q*^2^ values of 0.995, 0.502, and 0.293, respectively (Figure 5B). A small *Q*^2^ value indicates low predictive power of the model. Therefore, VIP values combined with fold-change values and *p-*values were used for identification. Forty differential metabolites were screened (Figures 5C,D). Among them, four differential metabolites belonged to nucleotides and their metabolomics. Additionally, two of each oxidized lipids, amines, coenzymes, and vitamins were identified. Lastly, one of each hormone and hormone-related compound, amino acid derivative, hydrocarbon derivative, DG, aldehyde, and MG were identified. In APP/PS1 mice, 31 metabolite concentrations were downregulated and 9 were upregulated. The top 10 differential metabolites according to the absolute value of log2FC were 9(S)-HpOTrE, acetoxy-8-gingerol, (8E,10S,12Z,15Z)-10-hydroperoxyoctadeca-8,12,15-trienoate, (-)-neplanocin A, 12-oxo-9(Z)-dodecenoic acid, terpenoid EA-I, excoecariatoxin;22,23,24,25-tetradehydro-simplexin, MG(22:5), forskolin, 2′-deoxyuridine, and 11-*cis*-retinol, as presented in Table 1.

**TABLE 1:** Differential metabolites and the relevant KEGG pathways.

Metabolites	Class II	KEGG pathway	A/C	VIP	Fold change	Log2FC	*P*
2′-deoxyuridine	Nucleotide and its metabolomics	Pyrimidine metabolism, metabolic pathways, ABC transporters	↓	2.34	3.65	1.87	0.01
Deoxyguanosine	Nucleotide and its metabolomics	Purine metabolism, metabolic pathways, ABC transporters	↓	2.18	2.83	1.50	0.03
Thymidine	Nucleotide and its metabolomics	Pyrimidine metabolism, metabolic pathways	↓	2.17	2.44	1.29	0.02
Deoxycytidine	Nucleotide and its metabolomics	Pyrimidine metabolism, metabolic pathways, ABC transporters	↓	2.32	2.80	1.48	0.02
8-hydroxy-2-deoxyguanosine	Nucleotide and its metabolomics		↓	2.43	2.73	1.45	0.02
15-oxoETE [15-oxo-5Z,8Z,11Z,13E-eicosatetraenoic acid]	Oxidized lipids	Arachidonic acid metabolism	↑	2.52	0.28	−1.81	0.03
9(S)-HpOTrE	Oxidized lipids		↑	2.37	0.11	−3.21	0.05
Acetaminophen glucuronide	Amines		↓	2.14	2.58	1.37	0.02
Octadecyl monoenamide	Amines		↓	3.49	2.71	1.44	0.00
11-*cis*-retinol	Coenzyme and vitamins	Retinol metabolism	↓	2.09	3.64	1.86	0.02
Thiamine	Coenzyme and vitamins	Thiamine metabolism, metabolic pathways, ABC transporters, folding, sulfur relay system, vitamin digestion and absorption	↑	2.61	0.41	−1.27	0.00
18-hydroxycorticosterone	Hormones and hormone-related compounds	Steroid hormone biosynthesis, metabolic pathways, aldosterone synthesis and secretion	↓	2.21	2.35	1.23	0.00
*N*-acetylphenylalanine	Amino acid derivatives	Phenylalanine metabolism, metabolic pathways	↑	1.59	0.39	−1.34	0.05
18β-glycyrrhetinic acid	Hydrocarbon derivatives		↓	2.58	2.19	1.13	0.01
1-single palm essence	DG		↓	2.39	3.03	1.60	0.02
9-octadecenal	Aldehydes		↓	1.28	2.13	1.09	0.00
MG(22:5)	MG		↓	2.97	4.00	2.00	0.00
Bis(2-ethylhexyl) phthalate		Chemical carcinogenesis – receptor activation	↓	3.37	2.22	1.15	0.00
4a-carboxy-4b-methyl-5a-cholesta-8,24-dien-3b-ol		Steroid biosynthesis, metabolic pathways	↓	2.24	2.05	1.04	0.02
4alpha-formyl-4beta-methyl-5alpha-cholesta-8,24-dien-3beta-ol			↓	1.95	2.08	1.06	0.05
Arbutin		Glycolysis/gluconeogenesis	↓	2.21	3.30	1.72	0.02
Forskolin			↓	1.91	3.85	1.95	0.02
(–)-Neplanocin A			↓	2.16	5.34	2.42	0.03
Pro Phe Lys			↓	1.79	3.57	1.83	0.04
*N*-oleoylethanolamine		cAMP signaling pathway	↓	2.46	2.04	1.03	0.01
(8E,10S,12Z,15Z)-10-hydroperoxyoctadeca-8,12,15-trienoate			↑	2.21	0.15	−2.71	0.05
12-oxo-9(Z)-dodecenoic acid		Alpha-linolenic acid metabolism	↑	2.18	0.21	−2.25	0.04
Acetoxy-8-gingerol			↑	2.84	0.12	−3.01	0.03
Ala Ser Asn Asp Leu			↓	1.30	3.57	1.84	0.03
Arg Ile Glu Asp			↓	1.70	2.03	1.02	0.04
Arg Leu Glu			↓	2.70	2.04	1.03	0.00
Arg Ser Tyr			↓	3.47	2.00	1.00	0.00
Dihydro Isorescinnamine			↓	2.95	3.20	1.68	0.00
Glu Glu Leu Ile Thr			↓	2.26	2.27	1.18	0.02
Ile Lys Ala Arg			↑	1.70	0.41	−1.30	0.03
Pro Leu Ala			↓	2.28	2.64	1.40	0.01
Terpenoid EA-I; excoecariatoxin; 22,23,24,25-tetradehydro-simplexin			↑	1.53	0.25	−2.01	0.05
Tyr Ser Asn Arg			↓	2.63	3.51	1.81	0.02
(1E,3R,10R,11S)-14-(hydroxymethyl)-3,10-dimethyl-6-propan-2-yltricyclo[9.3.0.03,7]tetradeca-1,6-dien-9-ol			↓	1.91	2.04	1.03	0.02
2-[(E,7R,11R)-3,7,11,15-tetramethylhexadec-2-enyl]naphthalene-1,4-diol			↓	2.13	2.36	1.24	0.03

Differential metabolites were determined by VIP ≥ 1, fold-change ≥ 2 or ≤0.5, and *P* < 0.05. A/C, APP/PS1 mice compared with WT mice; ↑, upregulated; ↓, downregulated.

#### Pathway analysis of differential metabolites

**FIGURE 6:**
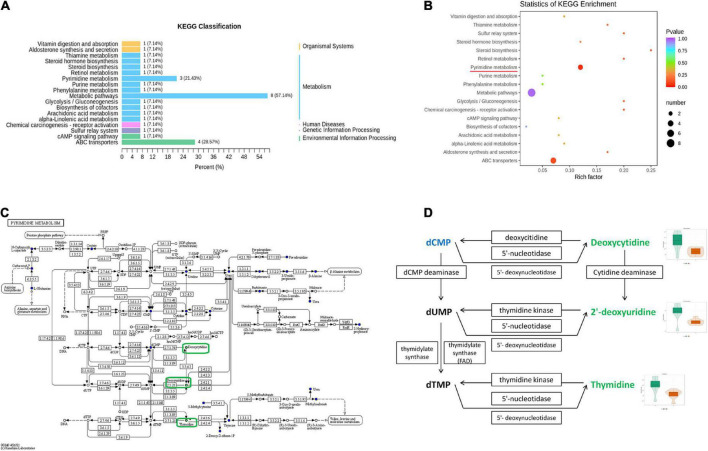
Pathway analysis of differential metabolites between APP/PS1 and wild-type mice. KEGG classification between the two groups **(A)** and significant KEGG enrichment of differential metabolites **(B)**. A diagram of pyrimidine metabolism in the KEGG pathway of APP/PS1 mice **(C)** and a detailed view of the three differential metabolites in it **(D)** (red, upregulated in APP/PS1 mice; green, downregulated in APP/PS1 mice; blue, the detected metabolites with no significant change). The violin plot shows the changes in the metabolites. Groups: A, APP/PS1 double-transgenic mice; C, wild-type control group of C57BL/6J mice; *n* = 8/group.

To further identify the relevant metabolic pathways involved in AD, differential metabolites were entered into the KEGG database for annotation, classification, and pathway enrichment analysis. The results showed that 57.14% of the differential metabolites were classified into metabolism (Figure 6A), containing 11 metabolic pathways in which pyrimidine metabolism was significantly perturbed (*P* = 0.044, Figures 6A,B). Three differential metabolites were enriched in the pyrimidine metabolic pathway, including deoxycytidine, 2′-deoxyuridine, and thymidine, all of which were depleted in APP/PS1 mice (Figure 6C). In the pyrimidine metabolic pathway, deoxycytidine is located upstream of 2′-deoxyuridine and thymidine, and they shared two common hydrolases, namely, 5′-nucleotidase and 5′-deoxynucleotidase (Figure 6D). ABC transporters contained four differential metabolites, but the differences between the groups were not significant (*P* = 0.109, Figures 6A,B).

### Correlation analysis of gut microbiota and fecal metabolites

**FIGURE 7:**
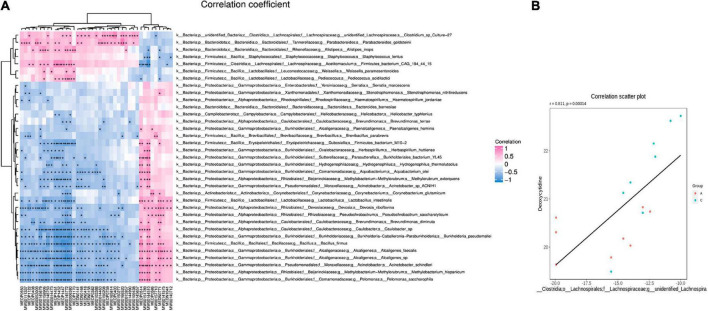
Correlation of gut microbiota and fecal metabolites. Heatmap summarizes the correlation between fecal metabolite alterations and gut microbiota perturbations at the species level in APP/PS1 and wild-type mice **(A)** (pink: positive correlation, blue: negative correlation). Scatter plot illustrates the statistical association between the relative abundance of *Clostridium* sp. Culture-27 and concentration of deoxycytidine **(B)**. Groups: A, APP/PS1 double-transgenic mice; C, wild-type control group of C57BL/6J mice; *n* = 8/group. *P < 0.05, **P < 0.01.

Based on the differences in gut microbiota and metabolites between the two groups, the functional correlation between them was investigated by calculating Spearman’s correlation coefficients. Associations between fecal metabolites and gut microbes were found at all bacterial taxonomic levels, and Figure 7A shows the correlations at the species level. A significant correlation was determined when *P* < 0.05, and | *r*| ≥ 0.8. Fourteen of the 40 differential metabolites were significantly correlated with the gut microbes (Table 2). *Bacillus firmus* and *Rikenella* were the top two bacteria related to most of the metabolites. *B. firmus* was negatively associated with five differential metabolites, while *Rikenella* was positively associated with four differential metabolites.

**TABLE 2:** Significant correlations between fecal metabolites and gut microbes.

Gut microbes	Differential metabolites	Index	*r*	*P*
*Bacillus firmus*	Arg Ser Tyr	MW00145772	–0.86	0.00
	Bis(2-ethylhexyl) phthalate	MW00006380	–0.82	0.00
	MG(22:5)	MEDP1452	–0.80	0.00
	Octadecyl monoenamide	MEDP1447	–0.89	0.00
	Thiamine	MEDP0514	0.81	0.00
*Rikenella*	11-*cis*-retinol	MEDP0408	0.81	0.00
	Bis(2-ethylhexyl) phthalate	MW00006380	0.83	0.00
	MG(22:5)	MEDP1452	0.86	0.00
	Octadecyl monoenamide	MEDP1447	0.83	0.00
*Erysipelotrichales*	18β-glycyrrhetinic acid	MEDP0759	–0.81	0.00
	Arg Ser Tyr	MW00145772	–0.81	0.00
*Acetitomaculum*	Acetoxy-8-gingerol	MW00144510	–0.89	0.00
	MG(22:5)	MEDP1452	0.80	0.00
*Alcaligenes faecalis*	15-oxoETE [15-oxo-5Z,8Z,11Z,13E-eicosatetraenoic acid]	MEDN0771	0.80	0.00
	Arg Ser Tyr	MW00145772	–0.82	0.00
*Alcaligenes* sp.	Arg Ser Tyr	MW00145772	–0.88	0.00
	Bis(2-ethylhexyl) phthalate	MW00006380	–0.83	0.00
*Burkholderia pseudomallei*	Bis(2-ethylhexyl) phthalate	MW00006380	–0.85	0.00
	MG(22:5)	MEDP1452	–0.81	0.00
*Firmicutes bacterium CAG 194_44_15*	Acetoxy-8-gingerol	MW00144510	–0.89	0.00
	MG(22:5)	MEDP1452	0.80	0.00
*Erysipelotrichaceae*	18β-glycyrrhetinic acid	MEDP0759	–0.81	0.00
	Arg Ser Tyr	MW00145772	–0.82	0.00
*Coriobacteriia*	9-octadecenal	MEDP1170	0.86	0.00
*Coriobacteriales*	9-octadecenal	MEDP1170	0.86	0.00
*Eggerthellaceae*	9-octadecenal	MEDP1170	0.86	0.00
*Enterorhabdus*	9-octadecenal	MEDP1170	0.82	0.00
*Alistipes*	MG(22:5)	MEDP1452	0.81	0.00
*Parabacteroides_goldsteinii*	2-[(E,7R,11R)-3,7,11,15-tetramethylhexadec-2-enyl]naphthalene-1,4-diol	MW00166520	0.89	0.00
*Bilophila*	9(S)-HpOTrE	MEDN1041	–0.82	0.00
*Candidatus_Stoquefichus*	(–)-Neplanocin A	MW00103388	0.89	0.00
*Dubosiella*	Arg Ser Tyr	MW00145772	–0.84	0.00
*Dolosigranulum*	Arg Ser Tyr	MW00145772	–0.81	0.00
*Caulobacterales*	Arg Ser Tyr	MW00145772	–0.81	0.00
*Caulobacteraceae*	Arg Ser Tyr	MW00145772	–0.81	0.00
*Gammaproteobacteria*	Acetoxy-8-gingerol	MW00144510	0.82	0.00
*Burkholderiales*	Acetoxy-8-gingerol	MW00144510	0.81	0.00
*Alcaligenaceae*	Arg Ser Tyr	MW00145772	–0.81	0.00
*Alcaligenes*	Arg Ser Tyr	MW00145772	–0.83	0.00
*Burkholderiaceae*	Bis(2-ethylhexyl) phthalate	MW00006380	–0.82	0.00
*Burkholderia-Caballeronia-Paraburkholderia*	Bis(2-ethylhexyl) phthalate	MW00006380	–0.82	0.00
*Comamonadaceae*	Arg Ser Tyr	MW00145772	–0.82	0.00
*Burkholderiales bacterium* YL45	Acetoxy-8-gingerol	MW00144510	0.81	0.00
*Clostridium* sp. Culture-27	Deoxycytidine	MEDP0403	0.81	0.00
*Coriobacteriia*	9-octadecenal	MEDP1170	0.83	0.00
*Coriobacteriales*	9-octadecenal	MEDP1170	0.83	0.00

Some microbiota-related metabolites were involved in specific KEGG pathways, including pyrimidine metabolism, ABC transporters, retinol metabolism, thiamine metabolism, folding, sulfur relay system, vitamin digestion and absorption, chemical carcinogenesis-receptor activation, and alpha-linolenic acid metabolism (Tables 1, 2). Notably, deoxycytidine, an important differential metabolite that causes alterations in pyrimidine metabolism, was positively correlated with *Clostridium* sp. Culture-27 (Figure 7B).

## Discussion

In this study, a combination of 16S rRNA gene sequencing and widely targeted metabolomics was utilized. To our knowledge, this is the first time the widely targeted metabolomics approach has been used in this field. We noted significant changes in gut microbiota and fecal metabolism of APP/PS1 mice compared with WT mice and a remarkable correlation between them. In particular, pyrimidine metabolism was found to be potentially involved in the pathology of AD, and the gut microbiota may contribute to this.

Cognitive deficit is a defining characteristic of AD. APP/PS1 mice are widely used in AD research (Wang M. et al., 2021) because they model well the pathological changes of AD such as senile plaque, neuronal deletion, amyloid-associated inflammation and cognitive decline. It was shown that APP/PS1 mice developed learning and memory deficits at 6 months of age (Webster et al., 2014), which is now considered to be the early stage of AD in this model. In the present study, APP/PS1 mice showed longer escape latencies in the place navigation test, indicating impaired learning abilities; however, the parameters in the spatial probe test did not vary significantly. In fact, the results are not entirely consistent across studies (Zheng et al., 2018; Luo et al., 2020), which may be related to different testing conditions and procedures. For example, the size of the pool can greatly affect the difficulty of the water maze task, and the swimming time in the probe test is also a factor in the outcome, as studies have shown that mice quadrant preference decreases within 30 s (Vorhees and Williams, 2006). The smaller pool used in this study (80 cm diameter vs. 120 cm diameter) and the longer swimming time (60 s vs. 30 s) of the probe test may mask mild memory impairment in mice.

Growing evidence links changes in gut microbiota to the etiology of AD (Chen et al., 2020) and suggests that microbiota-related metabolites play a vital role in microbiota-host interactions (Wu S. et al., 2021). In this study, we confirmed the lower β-diversity and increased *Firmicutes/Bacteroidota* ratio in AD, which is considered a marker of cognitive aging (Ticinesi et al., 2019). Multiple differential gut microbiota and fecal metabolites were identified. In agreement with other studies, beneficial bacteria such as *Candidatus saccharimonas* (Chen et al., 2017) and *Rikenellaceae* (Sun et al., 2020; Lee et al., 2021) decreased in APP/PS1 mice. Also, inflammatory or Aβ-associated bacteria such as *Erysipelotrichaceae* (Bäuerl et al., 2018; Ticinesi et al., 2019) and *Proteobacteria* (Nagpal et al., 2019; Ticinesi et al., 2019; Nagu et al., 2021) increased in APP/PS1 mice, which play key roles in the pathogenesis of AD. Similarly, some differential metabolites have been reported to be AD-related, such as 8-hydroxy-2-deoxyguanosine, a marker of nucleic acid oxidation (Mecocci et al., 2018; Cioffi et al., 2021), and beneficial substances, including 15-oxoETE (Snyder et al., 2015), 12-oxo-9(Z)-dodecenoic acid (Yang et al., 2021), arbutin (Dastan et al., 2019), 18β-glycyrrhetinic acid (Wagle et al., 2018), and *n*-oleoylethanolamine (Xu et al., 2016). Here, several previously unidentified differential bacteria and fecal metabolites were discovered, and their roles in AD need to be explored in further studies. It is worth mentioning that some bacteria showed opposite trends compared to those in other studies, including *Lactobacillus reuteri* (Mu et al., 2018), *Alistipes* (Ticinesi et al., 2019; Xu et al., 2020), *Eryslpelotrichales* (Kim et al., 2020), *Gammaproteobacteria* (Liu et al., 2019), and *Burkholderiales* (Ticinesi et al., 2019). These results suggest that other environmental and host factors are involved in the association between gut microbiota and AD (Köhler et al., 2016). The discovery of stable and specific microbial markers for AD requires additional studies and approaches.

Functional predictions of the differential microbiota and pathway analysis of differential metabolites can provide information on their function in disease pathology. Functional predictions revealed disturbances in AD-related pathways, including endocrine and metabolic diseases, aging, amino acid and nucleotide metabolism, energy, lipids, and vitamins. These results suggest that disturbances in certain metabolic pathways in APP/PS1 mice were probably driven by microbial alterations. Pathway analysis of differential fecal metabolites revealed a pyrimidine metabolism disorder in APP/PS1 mice. Pyrimidine biosynthesis is necessary for the maintenance of fundamental cellular functions and occurs in the mitochondria (Wang W. et al., 2021). Recently, researchers found pyrimidine metabolism disorder both in the cerebral cortex of Tg2576 mice (Dejakaisaya et al., 2021) and in the urine of demented rats (Huang et al., 2022), which, together with our findings, strongly suggests an association between pyrimidine metabolism and AD. In the present study, three differential metabolites were enriched in this pathway. Notably, deoxycytidine was located upstream of the other two metabolites, and all three metabolites were substrates of two hydrolases, namely, 5′-ribonuclease and 5′-deoxyribonuclease. Based on these results, we speculate that deoxyuridine may be the initiating link in the disorder of pyrimidine metabolism in APP/PS1 mice and that these two hydrolases may be the key substances involved. 5′-nucleotidases are catabolic members of the substrate cycle (Gazziola et al., 2001) and protect mitochondrial DNA replication from excess dTTP (Gallinaro et al., 2002). The simultaneous reduction of these three substrates suggests a possible decrease in the content or activity of hydrolases, including 5′-nucleotidases, which then leads to mitochondrial dysfunction, an early cellular change that plays a central role in AD pathology (Pradeepkiran and Reddy, 2020). Evidence suggests that defects in pyrimidine metabolism may translate into functional deficits during brain development (Fumagalli et al., 2017). Accordingly, we propose that affecting mitochondrial function is a way in which abnormal pyrimidine metabolism is involved in AD pathology and eventually leads to cognitive impairment.

Correlation analysis of gut microbiota and metabolites can provide broader ideas and additional information for AD marker screening and is superior to single-omics in understanding the role of gut microbiota in AD pathogenesis. The results showed that 40% of the fecal differential metabolites were significantly correlated with gut microbes. Furthermore, these microbiota-related metabolites are involved in pathways, such as vitamin or lipid metabolism, membrane transport, and genetic information processing, which are thought to participate in AD pathogenesis (Bai et al., 2020). *B. firmus* and *Rikenella* are related with most metabolites. These results strongly suggest an association between gut microbiota and fecal metabolites and that *B. firmus* and *Rikenella* may be important bacteria responsible for fecal metabolism alterations in APP/PS1 mice. Interestingly, a recent study found that *Rikenella* decreased in AD mice and was reversed in gut flora-targeted photobiomodulation therapy, indicating that *Rikenella* may be a potential therapeutic target for AD (Chen et al., 2021). Notably, deoxycytidine, an important differential metabolite that causes the pyrimidine metabolic alterations mentioned above, was positively correlated with *Clostridium* sp. Culture-27. A study identified the Na(+)-transporting NADH: ubiquinone reductase (NQR) in *Clostridium* sp. and concluded that its sequence could serve as a marker for monitoring AD risk, and it could be a new target for AD therapy (Paley et al., 2018). The present findings suggest that mutations in the gut *Clostridium* sp. Culture-27 may trigger abnormal pyrimidine metabolism in AD. These results indicate the involvement of gut dysbiosis in the disturbance of pyrimidine metabolism in APP/PS1 mice and highlight the interaction between host fecal metabolites and gut microbes in the pathological processes involved in AD. Metabolomic studies of serum and brain tissue or cerebrospinal fluid should be conducted to obtain more definitive conclusions.

This study has some limitations. First, it was a prospective study. The results were derived in part from bioinformatics and statistical analyses and have not been biologically validated. Second, this study was conducted using animal models, and the results require further validation in clinical trials.

## Conclusion

The gut microbiota may be involved in pathological processes associated with AD cognitive impairment by dysregulating pyrimidine metabolism. Several AD markers have been identified, particularly *B. firmus*, *Rikenella*, *Clostridium* sp. Culture-27, and deoxyuridine, which may play important roles in AD pathology. Our findings provide new insights into the host-microbe crosstalk in AD pathology and contribute to the discovery of diagnostic markers and therapeutic targets for AD. In addition, the roles of 5′-ribonuclease and 5′-deoxyribonuclease in pyrimidine metabolism perturbation in AD deserve further investigation. Finally, validating these findings in patients or postmortem studies is critical to translate them into clinical benefits.

## Data availability statement

The data of 16S rRNA gene sequencing presented in this study are deposited in the NCBI Sequence Read Archive (SRA) repository, accession number PRJNA857164, https://www.ncbi.nlm.nih.gov/bioproject/PRJNA857164. Other datasets used and/or analyzed in this study are available from the corresponding author on reasonable request.

## Ethics statement

The animal study was reviewed and approved by Ethics Committee of Hunan University of Medicine.

## Author contributions

MF, YZh, CZ, and QL conceived and designed the study. MZ, QC, and TY conducted the experiments. TH and QL performed the analyses. JB and YZe helped to analyze the data. MF and YZh wrote the manuscript. All authors have read and approved the final manuscript.
